# Clinical management protocols for community pharmacist-led management of urinary tract infections: a review of the grey literature and quality appraisal

**DOI:** 10.1007/s11096-024-01768-0

**Published:** 2024-07-15

**Authors:** Mitchell Budden, Daniel Gilbertson, Sean Chung, Shalom I. Benrimoj, Francisco Mardones, Sarah Dineen-Griffin

**Affiliations:** 1 College of Health, Medicine and Wellbeing, School of Biomedical Sciences and Pharmacy, University of Newcastle, University Drive, Callaghan, NSW 2308 Australia; 2 Deloitte Consulting, Melbourne, VIC Australia; 3 Pharmaceutical Care Research Group, Faculty of Pharmacy, University of Granada, Granada, Spain

**Keywords:** Clinical management protocol, Community pharmacist, Community pharmacy services, Evidence-based pharmacy practice, Urinary tract infections

## Abstract

**Background:**

Pharmacist-led management of urinary tract infections has been introduced as a service in the United Kingdom, Canada, United States of America, New Zealand, and Australia. The management of acute uncomplicated urinary tract infections by community pharmacists has gained increasing attention as a potential avenue to alleviate the burden on primary healthcare services.

**Aim:**

The objectives of the review were to: (1) identify protocols for community pharmacist management of acute uncomplicated urinary tract infections in women aged 16–65 years; (2) outline their key components; and (3) appraise the quality of protocols.

**Method:**

A grey literature search was undertaken for protocols intended for use by community pharmacists for the management of acute uncomplicated urinary tract infections in women aged 16–65 years, met the definition of a clinical management protocol and written in English. Their quality was appraised using the Appraisal Guidelines for Research and Evaluation version II instrument.

**Results:**

Forty of the 274 records screened were included. Content analysis identified ten key components: common signs/symptoms, differential diagnosis, red flags/referral, choice of empirical antibiotic therapy, nonprescription medications, nonpharmacological/self-care advice, patient eligibility criteria, patient follow-up, dipstick testing recommendations, and recommendations on antimicrobial resistance. The lowest scoring domains in the quality assessment were ‘Editorial Independence’ and ‘Rigour of Development’. Only four protocols were deemed high-quality.

**Conclusion:**

The review demonstrates that clinical management protocols for pharmacist-led management of urinary tract infections consist of similar recommendations, despite variation in international practice. However, the findings highlight a deficiency in the quality of most clinical management protocols governing pharmacist-led urinary tract infection management.

**Supplementary Information:**

The online version contains supplementary material available at 10.1007/s11096-024-01768-0.

## Impact statements

- Pharmacist-led management of urinary tract infections in women aged 16–65 years has become usual practice for community pharmacists in several countries.
- Implementing a standardised clinical management protocol to assist community pharmacists in the management of acute uncomplicated urinary tract infections could potentially reduce variability in care delivery, ensuring that patients receive consistent and evidence-based treatment regardless of location or health provider.
- The adoption of clinical management protocols streamlines decision-making processes for community pharmacists, which may enhance clinical efficiency and allow for more effective use of resources.
- Developers should use the Appraisal Guidelines for Research and Evaluation version II instrument as a framework to ensure comprehensiveness of their protocols.

## Introduction

Urinary Tract Infections (UTIs) are experienced by 10–15% of women each year [1]. UTIs occur more frequently in women than men, with one in three women and one in 20 men developing a UTI at some point in their lifetime [2, 3]. UTIs may be classified as uncomplicated (occur in a structurally and functionally normal urinary tract), or complex (occur in an abnormal urinary tract or in the presence of other complicating factors) [3].

A meta-analysis by Bent et al. [4] found that women who presented with at least two symptoms of a UTI (i.e., dysuria, urinary frequency, or urinary urgency) and the absence of vaginal discharge, had an approximately 90% probability of having an acute uncomplicated UTI [4–8]. In most females aged less than 65 years without complicating factors, a lower UTI can be reliably diagnosed according to the clinical presentation alone, without additional urinalysis [4–8]. A randomised controlled trial by Little et al. [9] comparing five different treatment approaches in the management of UTIs found that there was no evidence that either using midstream urine analysis as an initial strategy to guide antibiotic prescribing or the use of midstream urine samples by medical officers as part of their overall clinical management, improved patient symptoms or outcomes, including symptom intensity and duration [9].

Empirical antibiotic therapy for acute uncomplicated UTIs currently remains standard practice for managing most patients presenting with UTIs [4–8]. In many countries, access to antibiotics still requires patients to visit their GP for an assessment and prescription.

Internationally, there were over 1.8 million hospital admissions involving UTIs between 2018–19 and 2022–23 in the United Kingdom (UK) [10], 12.6% of female patients per year self-reported history of physician diagnosis of one or more UTIs in Canada [11], and in 2007, UTIs represented 0.9% of all ambulatory visits in the United States of America (USA) [11]. In Australia, UTIs accounted for 1.2% of all GP consultations in 2015–16 [12], 109,612 ED presentations in 2019–20 [13], and 76,854 hospitalisations for kidney infections and UTIs in 2017–18 [14].

Emerging evidence from several countries suggests that community pharmacists are well positioned to facilitate the assessment and management of acute uncomplicated UTIs in certain cohorts within the community pharmacy setting [15–17]. It has become usual practice in regions of the UK [6, 18–36], most provinces in Canada [15, 16, 37–40], some states in the USA [41–44], New Zealand (NZ) [45–47], and in some states across Australia [5, 7, 8, 48–53]. Pharmacist competencies and accessibility though opening times, convenient locations and usually no requirement for an appointment means that community pharmacists are well placed to support patients [1, 54].

To support the implementation of expanded scope services in community pharmacy, it is vital that community pharmacists are provided with appropriate tools and education, including clinical management protocols (CMPs). The development and agreement on the content of CMPs for the management and referral of acute uncomplicated UTIs is a process that ensures clinical governance, clinical guidance, and a standardised approach to managing patients. The definition of a CMP can be challenging, as it encompasses various aspects that can be difficult to encapsulate in a single definition [55–57]. The definition used in this paper acknowledges the multifaceted nature of such protocols. A ‘clinical management protocol’ is defined as “*a systematically developed algorithm or care pathway that incorporates evidence-based guidelines into a practical framework for decision making, thereby providing health practitioners with assistance in managing specific clinical circumstances”* [55–57]. CMPs offer advantages in the standardisation of care, aiming at improving clinical efficiency, reducing variability in practice, enhancing research opportunities, creating a cost-effective diagnosis/treatment algorithm, and ensuring that patients are treated with the appropriate level of care and safety [57–59].

### Aim

The objectives of the review were to: (1) identify protocols for community pharmacist management of acute uncomplicated urinary tract infections in women aged 16–65 years; (2) outline their key components; and (3) appraise the quality of the protocols.

## Method

The methodology included two phases: (1) a grey literature review conducted on March 13, 2024, searching for CMPs for pharmacist-led UTI management, and (2) performing a quality appraisal using the Appraisal Guidelines for Research and Evaluation (AGREE) version II instrument.

### Phase 1: literature review

#### Information sources and searching strategies

The grey literature search plan incorporated 3 search strategies: (1) customised Google search engines, (2) targeted websites, and (3) consultation with pharmacists working internationally. These strategies were adapted from those used by Godin et al. for applying systematic review search methods to grey literature [60].

Initially, CMPs were identified by searching Google with a range of phrases. From this initial process, a single key search strategy was devised to locate all or most of the management protocols of interest (Table 1).

**Table 1:** Search strategy

("pharmacies" OR "pharmacist" OR "community pharmacist" OR "community pharmacy" OR "pharmacy") AND ("urinary tract infections" OR "urinary tract infection" OR "UTI") AND (guidelines OR “clinical guidelines” OR “protocol” OR “PGD” OR “Patient Group Direction”)	

The second search strategy involved browsing targeted websites of relevant health organisations and agencies. A Google search was conducted to identify the relevant organisations and websites. Each of the relevant website homepages were ‘hand-searched’ for potentially relevant documents (e.g. web pages, reports).

The third search technique involved contacting professional organisations in the UK, NZ and Canada for additional protocols that may have been excluded from the search due to them being hidden behind the front website pages of professional organisations.

#### Eligibility assessment and study selection

**Fig. 1:**
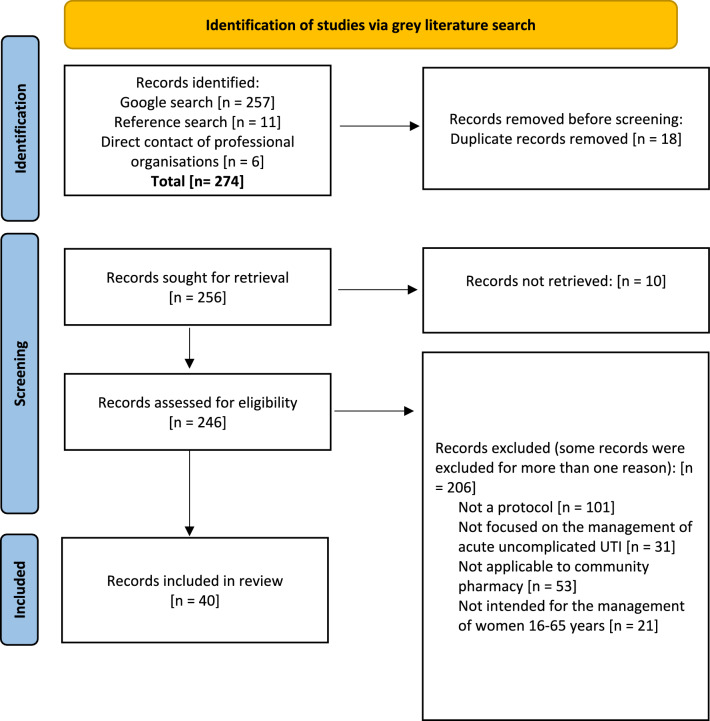
PRISMA like diagram of grey literature

The Preferred Reporting Items for Systematic Reviews and Meta-Analyses (PRISMA) process was applied to the grey literature search methods (Fig. 1). The title and source organisation of the identified documents were entered into an Excel sheet, and duplicates were removed. The titles of all search ‘hits’ were reviewed, a step that is analogous to a title screen in a traditional review of peer-reviewed literature. Being overinclusive, titles that appeared relevant were retained for further screening. The full text of all items, following the initial screening, were reviewed. Links were screened by title against the eligibility criteria by one reviewer (MB). Records were included in the review if they: (a) met the definition of a clinical management protocol; (b) were available in English; and (c) were intended for use by community pharmacists for the management of acute uncomplicated UTIs in women aged 16–65 years. Records were excluded if they did not comply with the inclusion criteria. When it was unclear whether an item met the eligibility criteria during screening, the reviewer was over-inclusive, and was taken for discussion with an additional two reviewers (SDG, SB). Reference lists of the included protocols were searched for any other potential protocols of interest [7, 22–27, 31–33]. Protocols pertaining to the management of complicated UTIs by health professionals other than community pharmacists were excluded, consistent with the primary objectives of the review.

#### Data extraction and synthesis

Following review of each publication, data pertaining to the source organisation, year published, by whom they were developed, intended audience, and country/region where the protocol is used was extracted. The content for CMPs underwent analysis for key components, with the component marked as present (✓), absent (✖) or partially mentioned (✓/✖).

### Phase 2: quality assessmet

Each CMP in the review was appraised by one reviewer (MB) using the AGREE II instrument. Any doubts were resolved with two other reviewers (SDG, SB). The AGREE II instrument is an appraisal tool designed to assess the quality of protocols and is considered the gold standard for appraisal in the international literature [61]. It comprises six domains and 23 items [61]. The AGREE II consortium has not set minimum domain scores or patterns of scores across domains to differentiate between high-quality and poor-quality protocols [61]. CMPs were deemed high-quality if the ‘Rigour of Development’ domain and if an additional 3 or more domains each scored ≥ 60% [61]. A protocol was recommended with modification if 3 or more domains each generated a score ≥ 50%, but not for the domain ‘Rigour of Development’ [61].

## Results

### Study selection

The search of grey literature yielded 257 potentially relevant items for screening. An additional 11 potentially relevant items were identified from reference lists during screening and a further six were obtained through communication with pharmacists internationally. Following elimination of duplicates, 246 unique records underwent screening based on their titles (Fig. 1). After a comprehensive review of the full texts, 40 CMPs met the criteria for inclusion in the review (Table 2). Nine of the protocols were published in Australia [5, 7, 8, 48–53], 19 in the UK [6, 19–36], five in Canada [16, 37–40], four in the USA [41–44], and three in New Zealand [45–47].

**Table 2:** Summary of included clinical management protocols by date

Title	Publication year	Publisher/Organisation	Country/Region
Patient Group Direction (PGD) Supply of Nitrofurantoin for uncomplicated Lower Urinary Tract Infections in females aged 16–64 years via the Pharmacy First Service (April 2023–April 2025) [28]	2024	Health and social care	United Kingdom
Patient Group Direction (PGD) Supply of Trimethoprim for uncomplicated Lower Urinary Tract Infections in females aged 16–64 years via the Pharmacy First Service (April 2023–April 2025) [29]	2024	Health and social care	United Kingdom
SA Community Pharmacy Urinary Tract Infection (UTI) Services: UTI Management Protocol [49]	2024	Government of South Australia, SA Health	Australia
Protocol for management of urinary tract infections: tasmanian community pharmacist pilot program [53]	2024	Tasmanian government, Department of health	Australia
Patient group direction (PGD) supply of nitrofurantoin capsules/tablets for the treatment of urinary tract infection (UTI) under the NHS England commissioned pharmacy first service [30]	2024	NHS England	United Kingdom
Pharmacist prescribing protocol: uncomplicated cystitis [39]	2024	Nova Scotia health authority	Canada
Pharmacist protocol for testing and initiating treatment for suspected acute uncomplicated lower urinary tract infection in women [41]	2023	Virginia board of pharmacy	United States of America
Protocol for management of urinary tract infections: victorian community pharmacist statewide pilot [51]	2023	Victoria state government Safe care victoria	Australia
Urinary tract infection treatment summary [48]	2023	The pharmacy guild of Australia WA branch	Australia
PATH-UTI pathway to access: UTI management clinical management protocol (main evaluation trial) management of urinary tract infections by community pharmacists [50]	2023	University of Newcastle	Australia
Community pharmacy UTI PGDs- summary flow chart [22]	2023	NHS Bath and Northeast Somerset, Swindon and Wiltshire integrated care board	United Kingdom
Protocol for testing and initiation of therapy for suspected acute uncomplicated lower urinary tract infection in women [43]	2023	Kansas State board of pharmacy	United States of America
Pharmaceutical society of new Zealand: nitrofurantoin for UTI algorithm [47]	2023	Pharmaceutical society of New Zealand	New Zealand
Pharmaceutical society of new Zealand: trimethoprim for UTI algorithm [46]	2023	Pharmaceutical society of New Zealand	New Zealand
Supply of trimethoprim tablets for the treatment of urinary tract infection (UTI) as part of the Hertfordshire and west Essex ICB community pharmacy infection management service [25]	2023	NHS Hertfordshire and West Essex	United Kingdom
NHS pharmacy first Scotland: national patient group direction (PGD) supply of nitrofurantoin tablets version 2.0 [31]	2022	NHS Scotland	United Kingdom
NHS pharmacy first Scotland: national patient group direction (PGD) supply of trimethoprim tablets version 2.0 [32]	2022	NHS Scotland	United Kingdom
Patient group direction (PGD) for administration/supply by pharmacists of trimethoprim 200 mg tablets for the treatment of uncomplicated urinary tract infections (UTI) in non-pregnant women on the isle of Wight [23]	2022	NHS Hampshire and Isle of Wight	United Kingdom
Pharmacist treatment guidance: uncomplicated cystitis [52]	2022	Australasian College of pharmacy	Australia
Urinary tract infection pharmacy pilot- Queensland: the management of urinary tract infections by community pharmacists: a state-wide trial [8]	2022	Queensland University of technology	Australia
Patient group direction (PGD) for the supply of nitrofurantoin 100 mg modified release capsules or nitrofurantoin 50mg tablets/capsules by registered pharmacists for the treatment of uncomplicated lower urinary tract infections in non-pregnant women [21]	2022	NHS South Sefton Clinical commissioning group NHS Southport and form by clinical commissioning group	United Kingdom
urinary tract infections (UTIS)- an overview of lower UTI management in adults [45]	2021	Best practice advocacy centre New Zealand	New Zealand
Assessment & prescribing algorithm for uncomplicated urinary tract infection (cystitis) [37]	2021	Ontario college of pharmacists Public health Ontario	Canada
The supply of nitrofurantoin 100 mg modified release capsules for the treatment of uncomplicated lower urinary tract infection in women by community pharmacists participating in the NHS Cheshire clinical commissioning group pharmacy first minor ailments service [24]	2021	NHS Cheshire clinical commissioning group	United Kingdom
The supply of Trimethoprim 200 mg tablets for the treatment of uncomplicated lower urinary tract infection in women (to be supplied when nitrofurantoin and Pivmecillinam are contraindicated or unavailable) by community pharmacists participating in the NHS Cheshire clinical commissioning group pharmacy first minor ailments service [35]	2021	NHS Cheshire clinical commissioning group	United Kingdom
The supply of Pivmecillinam 200 mg tablets for the treatment of uncomplicated lower urinary tract infection in women (to be supplied when nitrofurantoin is contraindicated or unavailable) by community pharmacists participating in the NHS Cheshire clinical commissioning group pharmacy first minor ailments service [34]	2021	NHS Cheshire clinical commissioning group	United Kingdom
How can you help your patients with an uncomplicated UTI? [38]	2021	Canadian pharmacists association	Canada
For the supply of Trimethoprim 100 mg or 200 mg tablets by registered pharmacists for the treatment of uncomplicated urinary tract infection in women under the Liverpool clinical commissioning group minor ailments service [26]	2021	NHS Liverpool	United Kingdom
SIGN160 Management of suspected bacterial lower urinary tract infection in adult women: a national clinical guideline [6]	2020	Scottish intercollegiate guidelines network (SIGN)	United Kingdom
Diagnosis of urinary tract infections: quick reference tool for primary care for consultation and local adaptation [20]	2020	public health England	United Kingdom
Pharmaceutical society of Australia: treatment guideline for pharmacists cystitis [5]	2020	Pharmaceutical society of Australia	Australia
Patient group direction for the supply of trimethoprim tablets by community pharmacists under the 'pharmacy first' service [27]	2020	NHS Grampian	United Kingdom
Acute, uncomplicated urinary tract infection treatment protocol V2 [42]	2019	Kentucky board of pharmacy	United States of America
Therapeutic guidelines: acute cystitis in adults [7]	2019	Therapeutic guidelines	Australia
NICE guideline- UTI (lower): antimicrobial prescribing [19]	2018	National institute for health and care excellence	United Kingdom
Treating your infection- urinary tract infection (UTI): for women under 65 years with suspected lower urinary tract infections (UTIs) or lower recurrent UTIs (cystitis or urethritis) for community pharmacy [33]	2017	NHS	United Kingdom
The assessment and management of urinary tract infections in adults: guidelines for pharmacists [16]	2017	Canadian pharmacists journal	Canada
Prince Edward island college of pharmacy. practice directive prescribing of drugs by pharmacists [40]	2014	Prince Edward Island college of pharmacy	Canada
Antibiotic guidelines for the management of infection in primary care 2013: uncomplicated lower UTI in women [36]	2013	NHS coastal west Sussex clinical commissioning group	United Kingdom
International clinical practice guidelines for the treatment of acute uncomplicated cystitis and pyelonephritis in women: a 2010 update by the infectious diseases society of America (IDSA) and the European society for microbiology and infectious diseases [44]	2011	Clinical infectious diseases	United States of America

### Clinical management protocol components

The content analysis yielded ten components which included: (1) common signs/symptoms, (2) differential diagnosis, (3) red flags/referral, (4) choice of empirical antibiotic therapy, (5) nonprescription medications, (6) nonpharmacological/ self-care advice, (7) patient eligibility criteria, (8) patient follow-up, (9) dipstick testing recommendations, and (10) recommendations on antimicrobial resistance (AMR) (Table 3). The degree of detail varied across protocols (see supplementary material 1 for the detailed extraction table).

**Table 3:** Components of clinical management protocols included in the review

Components	Present	Absent	Partially covered
Common signs/symptoms	38 protocols [5–8, 16, 20–24, 26, 27, 30, 33–40, 44–47, 49–53]	1 protocol [19]	1 protocol [48]
Differential diagnosis	22 protocols [5, 6, 8, 16, 20, 22, 24, 26, 30, 33–36, 38, 44–47, 50–53]	15 protocols [21, 23, 25, 27–32, 40–43, 48, 49]	3 protocols [19, 37, 39]
Red flags/referral	36 protocols [5, 8, 16, 19, 21–35, 37–53]	2 protocols [6, 36]	2 protocols [7, 20]
Choice of empirical antibiotic therapy	38 protocols [5–8, 16, 19, 21–32, 34–53]	1 protocol [33]	1 protocol [20]
Nonprescription medication	26 protocols [5–8, 19–24, 26, 27, 30–35, 38, 45–47, 50–53]	13 protocols [16, 25, 28, 29, 36, 37, 39–44, 48]	1 protocol [49]
Nonpharmacological/self-care advice	32 protocols [5, 6, 8, 19–35, 38, 39, 41, 43, 45–47, 49–53]	8 protocols [7, 16, 36, 37, 40, 42, 44, 48]	N/A
Patient eligibility criteria	36 protocols [5, 6, 8, 16, 19–35, 38–43, 46–53]	4 protocols [7, 36, 37, 44]	N/A
Patient follow-up	33 protocols [5, 6, 8, 16, 19–21, 23–30, 33–35, 37–43, 45–47, 49–53]	7 protocols [7, 22, 31, 32, 36, 44, 48]	N/A
Dipstick testing recommendations	9 protocols [6, 25, 28, 29, 31, 32, 41–43]	27 protocols [5, 7, 8, 16, 19, 21–24, 26, 27, 30, 33–35, 37–40, 44, 46, 47, 49–53]	4 protocols [20, 36, 45, 48]
Recommendations on AMR	4 protocols [8, 33, 44, 52]	33 protocols [5, 7, 19, 21–32, 34–36, 39–43, 45–53]	3 protocols [6, 16, 20]

Overall, the ten identified components were found to be present within most of the CMPs, with 36 of 40 management protocols demonstrating the presence of ≥ 5 components [5, 6, 8, 16, 19, 21–32, 34, 35, 38–43, 45–53]

The component *‘common sign/symptoms’* was one of the most comprehensively covered across the CMPs, with all but two CMPs covering comprehensively [19, 48].

For the component *‘differential diagnosis’,* three CMPs were reported as partially satisfying the component as they mentioned that a differential diagnosis should be conducted but failed to provide specific examples of conditions that may have overlapping symptoms with UTIs [19, 37, 39]. Similarly, the component *‘red flags/referral’* had two CMPs that were reported as partially satisfying the component as they did list the commonly associated red flags; however, they failed to differentiate these symptoms [7, 20].

For ‘*choice of empirical antibiotic therapy’,* all but two CMPs covered the component [20, 33]. One of the CMPs partially satisfied the component as it alluded to the use of antibiotics but did not provide specific recommendations for empirical antibiotic treatment [20].

For the component *‘nonprescription medications’,* 26 CMPs provided recommendations on managing UTIs using nonprescription medicines such as urinary alkalisers and anti-inflammatory medication (e.g., ibuprofen) [5–8, 19–24, 26, 27, 30–35, 38, 45–47, 50–53]. For the component *‘nonpharmacological/self-care advice’,* 32 CMPs provided nonpharmacological/self-care advice for the management of UTIs, such as increasing fluid intake [5, 6, 8, 19–35, 38, 39, 41, 43, 45–47, 49–53]. Similarly, the component *‘Patient eligibility criteria’* had 36 CMPs, which outlined eligibility criteria for patients to receive the UTI service in a community pharmacy [5, 6, 8, 16, 19–35, 38–43, 46–53]. For the component *‘patient follow-up',* 33 CMPs provided advice about the timing of patient follow-up [5, 6, 8, 16, 19–21, 23–30, 33–35, 37–43, 45–47, 49–53]. Seven CMPs did not provide any follow-up information [7, 22, 31, 32, 36, 44, 48].

For the component *‘dipstick testing recommendations’,* nine clinical management protocols discussed detailed dipstick testing recommendations [6, 25, 28, 29, 31, 32, 41–43]. Four CMPs were reported as partially satisfying the component as they suggested that dipstick testing could be used to support the diagnosis of a UTI but did not state this as a requirement of the protocol [20, 36, 45, 48]. The remaining 27 CMPs did not suggest that dipstick testing was required [5, 7, 8, 16, 19, 21–24, 26, 27, 30, 33–35, 37–40, 44, 46, 47, 49–53].

For the component *‘recommendations on AMR’,* three CMPs were reported as partially satisfying the component as they briefly mentioned AMR but did not specify strategies for minimising AMR [6, 16, 20]. Four CMPs provided specific recommendations on AMR [8, 33, 44, 52]. The remaining 33 CMPs did not provide any recommendations on AMR [5, 7, 19, 21–32, 34–36, 39–43, 45–53].

### Quality assessment

A total of 40 CMPs were evaluated using the AGREE II instrument. The appraisal scores ranged with a summary of the mean score for each domain reported in Table 4 (see supplementary material 2 for the detailed table) [5–8, 16, 19–53].

**Table 4:** Average score of the AGREE II domains across all protocols

Domain	Appraisal mean score (%)	Appraisal standard deviation score
1. Scope and Purpose	85.6	± 10.9
2. Stakeholder Involvement	42.6	± 17.5
3. Rigour of Development	16.7	± 19.3
4. Clarity of Presentation	68.1	± 7.1
5. Applicability	17.4	± 12.7
6. Editorial Independence	12.7	± 17.7

The domains with the lowest scores across all six AGREE II domains were ‘Editorial Independence’ (mean 12.7%, range 0–100%) and ‘Rigour of Development’ (mean 16.7%, range 2.1–83.3%), whereas the best performing domain was ‘Scope and Purpose’ (mean 84.6%, range 66.7–100%). The overall quality assessment of the included protocols varied. Only four protocols satisfied the criteria to be deemed high-quality [6, 19, 44, 50]. The National Institute for Health and Care Excellence (NICE), Scottish Intercollegiate Guidelines Network (SIGN160), International Clinical Practice Guidelines for the Treatment of Acute Uncomplicated Cystitis and Pyelonephritis in Women: A 2010 update by the Infectious Diseases Society of America and the European Society for Microbiology and Infectious Diseases (IDSA) and Pathway to access: UTI Clinical Management Protocol (PATH-UTI) protocols scored highly as they satisfied almost all of the required items within each domain. There were 21 protocols that scored above 50% [5, 7, 8, 16, 20, 21, 24, 28–32, 34, 35, 38, 45–47, 49, 51, 53]. The remaining 15 protocols were considered to be of lower quality as they scored below 50% [22, 23, 25–27, 33, 36, 37, 39–43, 48, 52].

## Discussion

### Statement of key findings

To our knowledge, this is the first international review to identify clinical management protocols for UTI management by community pharmacists, identify key components across protocols and undertake a quality appraisal. Capturing practice across a global landscape offers a comprehensive overview that extends beyond the confines of a singular healthcare system or region. The delineation between medical and pharmacy practice presents a challenge for community pharmacists managing UTIs, raising concerns about patient safety and pharmacist competency in expanding their scope of practice. Establishing collaborative, high-quality clinical protocols may help clarify this debate by defining the boundaries between professions and guiding their respective roles and responsibilities. In practice, CMPs provide a structured framework for pharmacists to assess and treat patients with acute uncomplicated UTIs. Most CMPs use an algorithmic approach, offering a step-by-step guide for the assessment and management of acute uncomplicated UTIs. If a patient does not meet the CMP criteria or a referral criterion is identified, the pharmacist is suggested to refer the patient to a medical practitioner for further evaluation and management. Some countries, such as Australia, have started using IT platforms that integrate their CMPs. This approach ensures adherence to CMPs by guiding pharmacists through each step and prompting the pharmacist when management or referral, or a combination, is required. The management approach may include the pharmacist prescribing empirical antibiotics. Additionally, most CMPs encourage pharmacists to provide non-pharmacological and self-care advice, including education about prevention for patients who may be at greater risk of developing recurrent UTIs.

### Strengths and weaknesses

Grey literature was the central source of information used for the identification of CMPs. Godin et al. acknowledge the challenges with applying systematic search methods to the grey literature, due to the lack of standards and resources for how to complete these searches [60]. The methodology utilised by Godin et al. has been employed in numerous previous studies [62–64]. This methodology enabled us to identify CMPs that could have been omitted if we had solely relied on academic sources of literature, such as protocols obtained from professional organisations that are not published in peer-reviewed databases [5, 6, 19, 39, 40, 46, 47, 50, 52]. A limitation of this review is the ability to access all available protocols for pharmacist-led UTI management. Despite contact with pharmacists practicing in Canada, we were only able to obtain protocols for specific provinces in Canada: New Brunswick, Nova Scotia, Ontario and Prince Edward Island. Another factor is the link between protocols and local resistance patterns and the legal status of available antibiotics. Although this is an important consideration, the primary focus of this review was identifying clinical management protocols for pharmacist-led UTI management. The AGREE II guideline suggests employing two to four reviewers to mitigate bias in evaluating the quality of CMPs. In this review, primarily one reviewer (MB) conducted the appraisal, with reference to two other reviewers (SDG, SB) in instances of uncertainty, and consultations were conducted until a consensus was achieved.

### Interpretation

Irrespective of country, this review demonstrates that the ten identified components were found to be present within most of the CMPs. Despite this, slight variation exists in relation to patient eligibility criteria, dipstick testing requirements and choice of empirical antibiotics. The practice implications that arise as a result of this variability could potentially compromise patient care and safety.

While there are similarities among CMPs despite variations in international practices, the findings underscore a deficiency in the quality of most CMPs governing pharmacist-led UTI management. The majority of CMPs exhibited insufficient reporting on stakeholder involvement (including consumers), not outlining rigor of development, insufficient provision of implementation information, and failure to declare editorial independence. The results coincide with previous findings from Alonso-Coello et al. which assessed a total of 626 clinical protocols using the AGREE II instrument [65]. Their study found that most protocols reported ‘Scope and Purpose’ (64%) and ‘Clarity of Presentation’ (60%) which is consistent with the results of this review [65]. It is evident that efforts should be made for protocol developers to undertake a methodological approach to searching the literature and documenting this information when developing protocols. Moreover, developers are advised to utilise the AGREE II tool as a framework, ensuring that their protocols comprehensively cover as many domains as possible. This approach has the potential to increase the likelihood of developing a high-quality protocol.

### Further research

The scarcity of peer-reviewed studies in this area underscores the need for ongoing research. Future reviews could potentially focus on the clinical outcomes of using CMPs in the delivery of standardised UTI management in community pharmacy. Additional research could focus on the link between protocols and local resistance patterns and the legal status of available antibiotics.

## Conclusion

A standardised CMP for community pharmacist-led management of UTIs, based on an assessment of existing literature and conceptualised with stakeholders, could be used to optimise practice. The preliminary findings and current evidence indicate that there are a large number of CMPs available for pharmacist-led UTI management. However, the content and quality of these protocols vary. To reduce fragmentation of primary care and enhance patient health outcomes, consistency between protocols must be prioritised.

## Supplementary Information

Below is the link to the electronic supplementary material.

Supplementary file1 (DOCX 41 KB)

Supplementary file2 (DOCX 38 KB)
