# Supplementary material for: Clinical management protocols for community pharmacist-led management of urinary tract infections: a review of the grey literature and quality appraisal
Source: Int J Clin Pharm. 2024 Jul 15;46(6):1256–67. doi: 10.1007/s11096-024-01768-0 (PMC11576775; doi:10.1007/s11096-024-01768-0)
Supplement: Supplementary file 1 — Supplementary file1 (DOCX 41 KB) [file 11096_2024_1768_MOESM1_ESM.docx]

**Supplementary Material 1: Components of clinical management protocols**

|  | **Publication year** | **Publisher/ Organisation** | **Country/**  **Region** | **Common signs/ symptoms** | **Differential diagnosis** | **Red flags/**  **referral** | **Choice of empirical antibiotic therapy** | **Nonprescription medications** | **Nonpharmacological/self-care advice** | **Patient eligibility criteria to receive the service** | **Patient follow-up** | **Dipstick testing recommendations** | **Recommendations on antimicrobial resistance** |
| --- | --- | --- | --- | --- | --- | --- | --- | --- | --- | --- | --- | --- | --- |
| Patient Group Direction (PGD) Supply of Nitrofurantoin for uncomplicated Lower Urinary Tract Infections in females aged 16 to 64 years via the Pharmacy First Service (April 2023 to April 2025) [28] | 2024 | Health and Social Care | United Kingdom | ✓ | ✖ | ✓ | ✓ | ✖ | ✓ | ✓ | ✓ | ✓ | ✖ |
| Patient Group Direction (PGD) Supply of Trimethoprim for uncomplicated Lower Urinary Tract Infections in females aged 16 to 64 years via the Pharmacy First Service (April 2023 to April 2025) [29] | 2024 | Health and Social Care | United Kingdom | ✓ | ✖ | ✓ | ✓ | ✖ | ✓ | ✓ | ✓ | ✓ | ✖ |
| SA Community Pharmacy Urinary Tract Infection (UTI) Services: UTI Management Protocol [49] | 2024 | Government of South Australia, SA Health | Australia | ✓ | ✖ | ✓ | ✓ | ✓/✖ | ✓ | ✓ | ✓ | ✖ | ✖ |
| Protocol for Management of Urinary Tract Infections: Tasmanian Community Pharmacist Pilot Program [53] | 2024 | Tasmanian Government, Department of Health | Australia | ✓ | ✓ | ✓ | ✓ | ✓ | ✓ | ✓ | ✓ | ✖ | ✖ |
| Patient Group Direction (PGD) Supply of Nitrofurantoin capsules/tablets for the treatment of Urinary Tract Infection (UTI) under the NHS England commissioned Pharmacy First service [30] | 2024 | NHS England | United Kingdom | ✓ | ✖ | ✓ | ✓ | ✓ | ✓ | ✓ | ✓ | ✖ | ✖ |
| Pharmacist Prescribing Protocol: Uncomplicated Cystitis [39] | 2024 | Nova Scotia Health Authority | Canada | ✓ | ✓/✖ | ✓ | ✓ | ✖ | ✓ | ✓ | ✓ | ✖ | ✖ |
| Pharmacist Protocol for Testing and Initiating Treatment for Suspected Acute Uncomplicated Lower Urinary Tract Infection in Women [41] | 2023 | Virginia Board of Pharmacy | United States of America | ✓ | ✖ | ✓ | ✓ | ✖ | ✓ | ✓ | ✓ | ✓ | ✖ |
| Protocol for Management of Urinary Tract Infections: Victorian Community Pharmacist Statewide Pilot [51] | 2023 | Victoria State Government Safe Care Victoria | Australia | ✓ | ✓ | ✓ | ✓ | ✓ | ✓ | ✓ | ✓ | ✖ | ✖ |
| Urinary Tract Infection Treatment Summary [48] | 2023 | The Pharmacy Guild of Australia WA Branch | Australia | ✓/✖ | ✖ | ✓ | ✓ | ✖ | ✖ | ✓ | ✖ | ✓/✖ | ✖ |
| PATH-UTI Pathway to access: UTI Management Clinical Management Protocol (Main Evaluation Trial) Management of Urinary Tract Infections by Community Pharmacists [50] | 2023 | University of Newcastle | Australia | ✓ | ✓ | ✓ | ✓ | ✓ | ✓ | ✓ | ✓ | ✖ | ✖ |
| Community Pharmacy UTI PGDs- Summary Flow Chart [22] | 2023 | NHS Bath and Northeast Somerset, Swindon and Wiltshire Integrated Care Board | United Kingdom | ✓ | ✓ | ✓ | ✓ | ✓ | ✓ | ✓ | ✖ | ✖ | ✖ |
| Protocol for Testing and Initiation of Therapy for Suspected Acute Uncomplicated Lower Urinary Tract Infection in Women [43] | 2023 | Kansas State Board of Pharmacy | United States of America | ✓ | ✖ | ✓ | ✓ | ✖ | ✓ | ✓ | ✓ | ✓ | ✖ |
| Pharmaceutical Society of New Zealand: Nitrofurantoin for UTI Algorithm [47] | 2023 | Pharmaceutical Society of New Zealand | New Zealand | ✓ | ✓ | ✓ | ✓ | ✓ | ✓ | ✓ | ✓ | ✖ | ✖ |
| Pharmaceutical Society of New Zealand: Trimethoprim for UTI Algorithm [46] | 2023 | Pharmaceutical Society of New Zealand | New Zealand | ✓ | ✓ | ✓ | ✓ | ✓ | ✓ | ✓ | ✓ | ✖ | ✖ |
| Supply of trimethoprim tablets for the treatment of urinary tract infection (UTI) as part of the Hertfordshire and West Essex ICB Community Pharmacy Infection Management Service [46] | 2023 | NHS Hertfordshire and West Essex | United Kingdom | ✓ | ✖ | ✓ | ✓ | ✖ | ✓ | ✓ | ✓ | ✓ | ✖ |
| NHS Pharmacy First Scotland: National Patient Group Direction (PGD) Supply of Nitrofurantoin Tablets Version 2.0 [31] | 2022 | NHS Scotland | United Kingdom | ✓ | ✖ | ✓ | ✓ | ✓ | ✓ | ✓ | ✖ | ✓ | ✖ |
| NHS Pharmacy First Scotland: National Patient Group Direction (PGD) Supply of Trimethoprim Tablets Version 2.0 [32] | 2022 | NHS Scotland | United Kingdom | ✓ | ✖ | ✓ | ✓ | ✓ | ✓ | ✓ | ✖ | ✓ | ✖ |
| Patient Group Direction (PGD) For administration/supply by pharmacists of Trimethoprim 200mg Tablets for the treatment of uncomplicated urinary tract infections (UTI) in non-pregnant women on the Isle of Wight [23] | 2022 | NHS Hampshire and Isle of Wight | United Kingdom | ✓ | ✖ | ✓ | ✓ | ✓ | ✓ | ✓ | ✓ | ✖ | ✖ |
| Pharmacist Treatment Guidance: Uncomplicated Cystitis [52] | 2022 | Australasian College of Pharmacy | Australia | ✓ | ✓ | ✓ | ✓ | ✓ | ✓ | ✓ | ✓ | ✖ | ✓ |
| Urinary Tract Infection Pharmacy Pilot- Queensland: The management of urinary tract infections by community pharmacists: A state-wide trial [8] | 2022 | Queensland University of Technology | Australia | ✓ | ✓ | ✓ | ✓ | ✓ | ✓ | ✓ | ✓ | ✖ | ✓ |
| Patient Group Direction (PGD) for the supply of Nitrofurantoin 100mg Modified Release Capsules or Nitrofurantoin 50mg tablets/capsules by registered pharmacists for the treatment of uncomplicated lower urinary tract infections in non-pregnant women [21] | 2022 | NHS South Sefton Clinical Commissioning Group NHS Southport and Formby Clinical Commissioning Group | United Kingdom | ✓ | ✖ | ✓ | ✓ | ✓ | ✓ | ✓ | ✓ | ✖ | ✖ |
| Urinary tract infections (UTIs)- an overview of lower UTI management in adults [45] | 2021 | Best Practice Advocacy Centre New Zealand | New Zealand | ✓ | ✓ | ✓ | ✓ | ✓ | ✓ | ✓ | ✓ | ✓/✖ | ✖ |
| Assessment & Prescribing Algorithm for Uncomplicated Urinary Tract Infection (cystitis) [37] | 2021 | Ontario College of Pharmacists Public Health Ontario | Canada | ✓ | ✓/✖ | ✓ | ✓ | ✖ | ✖ | ✖ | ✓ | ✖ | ✖ |
| The supply of Nitrofurantoin 100mg Modified Release Capsules for the treatment of uncomplicated lower urinary tract infection in women by community pharmacists participating in the NHS Cheshire Clinical Commissioning Group Pharmacy First Minor Ailments Service [24] | 2021 | NHS Cheshire Clinical Commissioning Group | United Kingdom | ✓ | ✓ | ✓ | ✓ | ✓ | ✓ | ✓ | ✓ | ✖ | ✖ |
| The supply of Trimethoprim 200mg Tablets for the treatment of uncomplicated lower urinary tract infection in women (to be supplied when Nitrofurantoin and Pivmecillinam are contraindicated or unavailable) by community pharmacists participating in the NHS Cheshire Clinical Commissioning Group Pharmacy First Minor Ailments Service [35] | 2021 | NHS Cheshire Clinical Commissioning Group | United Kingdom | ✓ | ✓ | ✓ | ✓ | ✓ | ✓ | ✓ | ✓ | ✖ | ✖ |
| The supply of Pivmecillinam 200mg Tablets for the treatment of uncomplicated lower urinary tract infection in women (to be supplied when Nitrofurantoin is contraindicated or unavailable) by community pharmacists participating in the NHS Cheshire Clinical Commissioning Group Pharmacy First Minor Ailments Service [34] | 2021 | NHS Cheshire Clinical Commissioning Group | United Kingdom | ✓ | ✓ | ✓ | ✓ | ✓ | ✓ | ✓ | ✓ | ✖ | ✖ |
| How can you help your patients with an uncomplicated UTI? [38] | 2021 | Canadian Pharmacists Association | Canada | ✓ | ✓ | ✓ | ✓ | ✓ | ✓ | ✓ | ✓ | ✖ | ✖ |
| For the supply of Trimethoprim 100mg or 200mg tablets by registered pharmacists for the Treatment of Uncomplicated Urinary Tract Infection in women under the Liverpool Clinical Commissioning Group Minor Ailments Service [26] | 2021 | NHS Liverpool | United Kingdom | ✓ | ✓ | ✓ | ✓ | ✓ | ✓ | ✓ | ✓ | ✖ | ✖ |
| SIGN160 Management of suspected bacterial lower urinary tract infection in adult women: A national clinical guideline [6] | 2020 | Scottish Intercollegiate Guidelines Network (SIGN) | United Kingdom | ✓ | ✓ | ✖ | ✓ | ✓ | ✓ | ✓ | ✓ | ✓ | ✓/✖ |
| Diagnosis of urinary tract infections: Quick reference tool for primary care for consultation and local adaptation [20] | 2020 | Public Health England | United Kingdom | ✓ | ✓ | ✓/✖ | ✓/✖ | ✓ | ✓ | ✓ | ✓ | ✓/✖ | ✓/✖ |
| Pharmaceutical Society of Australia: Treatment Guideline for Pharmacists Cystitis [5] | 2020 | Pharmaceutical Society of Australia | Australia | ✓ | ✓ | ✓ | ✓ | ✓ | ✓ | ✓ | ✓ | ✖ | ✖ |
| Patient Group Direction for the supply of Trimethoprim tablets by Community Pharmacists under the 'Pharmacy First' service [27] | 2020 | NHS Grampian | United Kingdom | ✓ | ✖ | ✓ | ✓ | ✓ | ✓ | ✓ | ✓ | ✖ | ✖ |
| Acute, uncomplicated urinary tract infection treatment protocol V2 [42] | 2019 | Kentucky Board of Pharmacy | United States of America | ✓ | ✖ | ✓ | ✓ | ✖ | ✖ | ✓ | ✓ | ✓ | ✖ |
| Therapeutic Guidelines: Acute cystitis in adults [7] | 2019 | Therapeutic Guidelines | Australia | ✓ | ✓ | ✓/✖ | ✓ | ✓ | ✖ | ✖ | ✖ | ✖ | ✖ |
| NICE Guideline- UTI (lower): antimicrobial prescribing [19] | 2018 | National Institute for Health and Care Excellence | United Kingdom | ✖ | ✓/✖ | ✓ | ✓ | ✓ | ✓ | ✓ | ✓ | ✖ | ✖ |
| Treating your infection- Urinary Tract Infection (UTI): For women under 65 years with suspected lower urinary tract infections (UTIs) or lower recurrent UTIs (cystitis or urethritis) For Community Pharmacy [33] | 2017 | NHS | United Kingdom | ✓ | ✓ | ✓ | ✖ | ✓ | ✓ | ✓ | ✓ | ✖ | ✓ |
| The assessment and management of Urinary Tract Infections in adults: Guidelines for pharmacists [16] | 2017 | Canadian Pharmacists Journal | Canada | ✓ | ✓ | ✓ | ✓ | ✖ | ✖ | ✓ | ✓ | ✖ | ✓✖ |
| Prince Edward Island College of Pharmacy. Practice Directive Prescribing of Drugs by Pharmacists [40] | 2014 | Prince Edward Island College of Pharmacy | Canada | ✓ | ✖ | ✓ | ✓ | ✖ | ✖ | ✓ | ✓ | ✖ | ✖ |
| Antibiotic Guidelines for the Management of infection in primary care 2013: Uncomplicated Lower UTI in Women [36] | 2013 | NHS Coastal West Sussex Clinical Commissioning Group | United Kingdom | ✓ | ✓ | ✖ | ✓ | ✖ | ✖ | ✖ | ✖ | ✓/✖ | ✖ |
| International Clinical Practice Guidelines for the Treatment of Acute Uncomplicated Cystitis and Pyelonephritis in Women: A 2010 update by the Infectious Diseases Society of America (IDSA) and the European Society for Microbiology and Infectious Diseases. [44] | 2011 | Clinical Infectious Diseases | United States of America | ✓ | ✓ | ✓ | ✓ | ✖ | ✖ | ✖ | ✖ | ✖ | ✓ |
